# Killing of Latently HIV-Infected CD4 T Cells by Autologous CD8 T Cells Is Modulated by Nef

**DOI:** 10.3389/fimmu.2018.02068

**Published:** 2018-09-11

**Authors:** Ziv Sevilya, Ehud Chorin, Orit Gal-Garber, Einat Zelinger, Dan Turner, Boaz Avidor, Gideon Berke, David Hassin

**Affiliations:** ^1^Internal Medicine Department A, Assuta Ashdod Medical Center, Ashdod, Israel; ^2^Crusaid Kobler AIDS center, Tel-Aviv Sourasky Medical Center, Tel-Aviv, Israel; ^3^Sackler Faculty of Medicine, Tel-Aviv University, Tel-Aviv, Israel; ^4^Interdepartmental Equipment Facility, Robert H. Smith Faculty of Agriculture, Food and Environment, the Hebrew University, Rehovot, Israel; ^5^Department of Immunology, Weizmann Institute of Science, Rehovot, Israel; ^6^Faculty of Health Sciences, Ben Gurion University of the Negev, Beer-Sheva, Israel

**Keywords:** CD8 T cells, cellular immunology, HIV, virology, latent reservoir HIV infected CD4 T cells, apoptosis of HIV infected CD4 T cells

## Abstract

The role of HIV-specific CD8 T cell activity in the course of HIV infection and the way it affects the virus that resides in the latent reservoir resting memory cells is debated. The PBMC of HIV-infected patients contain HIV-specific CD8 T cells and their potential targets, CD4 T cells latently infected by HIV. CD4 T cells and CD8 T cells procured from PBMC of HIV-infected patients were co-incubated and analyzed: Formation of CD8 T cells and HIV-infected CD4 T cell conjugates and apoptosis of these CD4 T cells were observed by fluorescence microscopy with *in situ* PCR of HIV LTR DNA. Furthermore, conjugation of CD8 T cells with CD4 T cells and apoptosis of CD4 T cells was observed and quantified by imaging flow cytometry using anti-human activated caspase 3 antibody and TUNEL assay. The conjugation activity and apoptosis were found to be much higher in patients with acute HIV infection or AIDS compared to patients in chronic infection on antiretroviral therapy (ART) or not. Patients on ART had low grade conjugation and apoptosis of isolated CD69, CD25, and HLA-DR-negative CD4 T cells (latent reservoir cells) by CD8 T cells. Using *in situ* PCR The latent reservoir CD4 T cells were shown to contain most of the HIV DNA. We demonstrate in HIV-infected patients, that CD8 T cells conjugate with and kill HIV-infected CD4 T cells, including HIV-infected resting memory CD4 T cells, throughout the course of HIV infection. We propose that in HIV-infected patients CD4 T cell annihilation is caused in part by ongoing activity of HIV-specific CD8 T cells. HIV Nef protein interacts with ASK 1 and inhibits its pro-apoptotic death signaling by Fas/FasL, thus protecting HIV-infected cells from CD8 T cells killing. A peptide that interrupts Nef-ASK1 interaction that had been delivered into CD4 T cells procured from patients on ART resulted in the increase of their apoptosis inflicted by autologous CD8 T cells. We suggest that elimination of the HIV-infected latent reservoir CD4 T cells can be achieved by Nef inhibition.

## Introduction

HIV-infected patients are currently treated by antiretroviral therapy (ART), which achieves viral suppression but not a cure. Latent reservoirs of resting memory HIV-infected CD4 T cells are established very early after infection and result in a viral rebound upon treatment interruption. The biology of latent reservoirs cells is poorly understood (1, 2). The CD8 T cell receptor (TCR) recognize a viral epitope presented in the HLA class 1 receptor, resulting in the formation of an immunological synapse between the CD8 T cells and the target cells, which can be observed as a CD8 T cell-target cell conjugate. This conjugate formation was found to be antigen and TCR specific; CD8 T cells conjugate with and kill only peptide-MHC restricted target cells that the immune system was previously exposed to (3–6). Immunological synapse formation results in the apoptosis of the target cell by the interaction of cell surface receptors, Fas-FasL, and by perforin and granzymes secreted from the CD8 T cells. Perforin expression in the CD8 T cells decays very rapidly upon the disappearance of the target cells, it can therefore serve as a marker of ongoing activation and killing capacity of CD8 T cells (3, 7). The role of CD8 T cells in the course of HIV infection and their possible effects on the latent reservoir of CD4 T cells is unknown.

The appearance of HIV-specific CD8 T cells in acute HIV infection correlates with the decline in both viral load and number of CD4 T cells (8–11). Depletion of CD8 T cells in simian immunodeficiency virus (SIV)-infected macaques resulted in a rapid rise in the plasma viral load (8, 11). The frequency of CD8 T cells that target multiple HIV proteins in the peripheral blood mononuclear cells (PBMC) of untreated HIV-infected patients is 8.7 ± 1.38% (12). Functional HIV-specific CD8 T cells can be identified in the PBMC of patients on prolonged ART with undetectable viral load (12–15). A broad HIV-specific CD8 T cell response against Gag and Nef proteins in ART-treated patients was observed recently; suggesting preservation of active CD8 T cells immune function (15, 16). These data imply that viral proteins are expressed and presented by resting memory CD4 T cells recognized by CD8 T cells.

PBMC of HIV-infected patients contain HIV-specific CD8 T cells and their potential targets, HIV-infected CD4 T cells. The frequency of cells harboring HIV pro-viral DNA in lymph nodes and gut biopsies is comparable to the frequency of chronic HIV-infected memory CD4 T cells in the PBMC of HIV infected patients (17). We previously studied the interaction between CD8 and CD4 T cells in untreated HIV-infected patients. CD4 and CD8 T cells were directly procured from the PBMC of acute and chronic untreated HIV-infected patients and co-incubated (the cells were neither manipulated nor stimulated *in vitro*). Formation of CD8-CD4 T cell conjugates and CD4 T cells apoptosis were observed by fluorescence microscopy and by continuous recording in tissue culture. It emerged that 6% of the CD4 T cells from acute HIV-infected patients were conjugated by autologous CD8 T cells, while 3% of the CD4 T cells were conjugated by autologous CD8 T cells in chronic untreated HIV-infected patients. Annexin binding and cell morphology typical of apoptosis were observed in the conjugated CD4 T cells. The majority of CD8 T cells that had conjugated to CD4 T cells expressed perforin. Apoptosis was significantly elevated in acute HIV infection and more modest during the chronic phase of untreated HIV infection. The CD4 T cells conjugated by autologous CD8 T cells procured from chronic untreated HIV-infected patients were positive for HIV DNA, as observed by *in situ* PCR (5).

It has been suggested that the HIV Nef protein may play an important role in the ability of HIV to evade the immune system (18). The HIV Nef protein down regulates HLA expression and protects HIV-infected cells from being killed by cytotoxic T lymphocytes (CTL) (19). Nef was associated with Apoptosis Signal regulating Kinase 1 (ASK1) which protected the Nef transfected CD4 T cells from apoptosis by FasL and TNF-α (20, 21).

We studied the interaction between CD8 and CD4 T cells procured from the PBMC of AIDS, acute, and chronic untreated and treated HIV-infected patients. The cells were studied by fluorescent microscopy, *in situ* PCR of HIV DNA and imaging flow cytometry. We found that CD8 T cells form conjugates and kill HIV-infected CD4 T cells in all stages of the infection, including in HIV-infected patients on ART. The conjugation activity and apoptosis rates were much higher in patients with acute infection or AIDS than in chronic untreated and treated patients. Most of the CD4 T cells from chronic and treated HIV-infected patients that were positive for HIV DNA by *in situ* PCR were resting memory cells. The autologous CD8 T cells were shown to conjugate with and kill latent reservoir CD4 T cells. A peptide that interrupts Nef-ASK1 interaction that had been delivered into CD4 T cells procured from patients on ART resulted in the increase of their apoptosis inflicted by autologous CD8 T cells.

## Materials and methods

### Study subjects

Twenty-eight HIV-infected patients in acute, chronic untreated, treated by ART and AIDS patients as well as 14 matched healthy controls were enrolled into this study at the Crusaid Kobler AIDS Center, Tel Aviv Sourasky Medical Center, Israel (Table 1). Acute HIV-infected patients were defined 3–12 weeks after clinical presentation. Chronic untreated HIV-infected subjects were defined as patients at least 1 year after HIV infection. AIDS patients were late presenters with CD4 T cell counts below 200 cell/μl. All the patients on ART had an undetectable viral load <20 copies/ml and a CD4 T cell count above 360 cell/μl. Plasma viral load and CD4 and CD8 T lymphocyte counts were determined as previously described (5). All subjects provided written informed consent for participation in the study, which was approved by the institutional ethics committee in accordance with the ethical standards laid down in the 1964 Declaration of Helsinki and its later amendments.

**Table 1:** Characteristics of the patients enrolled in this study.

**Case**	**Age**	**CD4 (cells/μl)**	**CD8 (cells/μl)**	**Viral load (copies/ml)**		
**ACUTE HIV INFECTED PATIENTS**						
1	26	609	2523	60148		
2	53	419	550	88088		
3	55	504	1584	876323		
**Case**	**Age**	**CD4 (cells/**μ**l)**	**CD8 (cells/**μ**l)**	**Viral load**		
**AIDS PATIENTS**						
1	32	84	1032	65540		
2	36	72	204	490565		
3	28	42	504	273074		
**Case**	**Age**	**CD4 (cells/**μ**l)**	**CD8 (cells/**μ**l)**	**Viral load (copies/ml)**	**Time from infection (years)**
**CHRONIC HIV INFECTED PATIENTS**						
1	32	624	656	2801	3	
2	34	713	897	989	2	
3	26	546	957	19611	1	
**Case**	**Age**	**CD4 (cells/**μ**l)**	**CD8 (cells/**μ**l)**	**Viral load**	**Time from infection (years)**	**Time on ART (years)**
**PATIENTS ON ANTIRETROVIRAL THERAPY**						
1	50	666	630	TND	8	8
2	63	506	546	TND	9	8
3	40	736	1664	TND	10	7
4	48	612	680	TND	7	4
5	45	624	1182	TND	13	9
6	48	738	360	TND	1	0.5
7	43	903	736	TND	6	4
8	51	408	799	TND	8	6
9	51	544	621	TND	20	17
10	49	945	588	TND	5	4
11	46	1131	1218	TND	8	6
12	42	1128	962	TND	7	6
13	26	850	1462	TND	1	0.5
14	42	750	1000	TND	2.5	2
15	44	507	390	TND	10	9
16	37	1131	986	TND	4	3
17	36	680	510	TND	5	4
18	43	1196	667	TND	16	15
19	25	1000	625	TND	3	2

### Isolation of CD8 T cells and CD4 T cells from PBMC

Thirty milliliters of whole blood were collected in EDTA and the PBMC were separated over a Ficoll-Hypaque density gradient. CD8 T cells were positively selected using CD8 MicroBeads kit (Miltenyi Biotech), according to the manufacturer's protocol. CD4 T cells were separated from the CD8 T cell-depleted PBMC by negative selection of CD4 T cells (CD4 T cell isolation kit, Miltenyi Biotech). Purity of the CD4 and CD8 T cells was tested by Imaging flow cytometer (ImageStream) and determined to be above 95%.

### Separating resting from activated CD4 T cells

Resting CD4 T cells were separated from activated CD4 T cells using a two-step negative selection method based on the activated cells surface markers CD25, CD69, and HLA-DR, (22–24) using CD69 MicroBead kit II, Anti-HLA-DR MicroBeads, and anti-human CD25 conjugated to microbeads (MicroBeads II, Miltenyi Biotech) according to the manufacturer's protocol (Miltenyi Biotech). The purity of the separated cells was demonstrated by staining with anti-CD69 (Enzo), anti-HLA-DR (Enzo), and anti-CD25 (Enzo) antibodies conjugated to APC and analyzed by the ImageStream. Typical purities of resting CD4 T cells were 95–98.5% with <1.5% contamination by activated CD4 T cells (**Figure 4**).

### Conjugate formation of CD8-CD4 T cells

CD4 T cells were labeled with 2 μM calcein AM (Molecular probes C1430). Freshly isolated 1 × 10^6^ CD4 and 1 × 10^6^ CD8 T cells were mixed in 1 ml PBS containing 0.9 mM CaCl_2_ and 0.5 mM MgCl_2_, incubated for 15 min at 37°C, and then co-centrifuged at 185 g for 20 min at room temperature. They were re suspended in cold PBS containing 0.9 mM CaCl_2_ and 0.5 mM MgCl_2_ and placed on ice. Conjugates of the CD8 T cells with CD4 T cells were immediately quantified in the hemocytometer by fluorescence microscopy (5). Conjugated CD4 and CD8 T cells were fixed in 4% PFA (15 min, 22°C). The fixed cells were incubated with 2% fetal bovine serum (FBS) in PBS (1 h, 4°C) followed by staining with anti-human CD8 antibody conjugated to APC (clone HIT8a, Biolegend). The slides were visualized by a confocal microscope. At least 1,000 cells were scored for each patient. The percent conjugation is the number of conjugated CD4 T cells divided by the total number of CD4 T cells X100. Counting CTL target cell conjugates under a microscope has been shown to be both accurate and specific (3–5).

### *In situ* PCR of HIV DNA

The method was adopted from the protocols published (5, 25–28). Following conjugation of CD4 T cells with CD8 T cells, 1 × 10^5^ cells were fixed with 4% PFA on slides and an *in-situ* PCR amplification reaction in a thermal cycler was performed for 30 cycles. The primers are from the HIV LTR: Forward primer (NEC 152) 5′-GCCTCAATAAAGCTTGCCTTGA-3′. Reverse primer (NEC 131) – 5′-GGCGCCACTGCTAGAGATTTT-3′ (27–29). After amplification, fluorescein-tagged 56nt probe was used to identify the amplified HIV DNA: 5′-CACAACAGACGGGCACACACCTACTTTAAGCACTCAAGGCAAGCTTTATTGAGGCA-3′ (5). When indicated, after the *in-situ* PCR the slides were labeled with anti-human perforin antibody (clone dG9 Biolegend 308109) conjugated to AlexaFlour 647. The slides were observed on a confocal microscope, and 1,000 cells were screened for cells harboring HIV DNA and CD4-CD8 T cell conjugates in each slide.

### Immunological synapse proteins in CD8-CD4 T cells conjugates

Conjugated CD8-CD4 T cells procured from patients on ART were fixed with 4% PFA, permeabilized and stained with anti-human CD8 antibody conjugated to PerCP/Cy5.5 (clone HIT8a, Biolegend), anti-human perforin antibody clone dG9 conjugated with B.V.510 (Biolegend), anti-human γ-tubulin (clone D-10, Santa Cruz) conjugated with PE, anti-human LCK antibody (Y123 RabMab, abcam) and observed by a confocal microscope.

### CD4-CD8 T cells conjugation analyzed by multispectral imaging flow cytometry (imagestream)

After conjugation, the cells were fixed with 4% PFA, they were incubated with anti-human CD8 antibody conjugated with APC (clone HIT8a, Biolegend), anti-human CD4 antibody PE/Cy7 conjugated (clone OKT, Biolegend) and 1.4 μM DAPI. The cells were imaged by a multispectral imaging flow cytometer (ImageStreamX flow-cytometer; Amnis Corp., Seattle, WA). A total of 2–5 × 10^4^ cells were collected from each sample and the data were analyzed using image analysis software (IDEA 6.0; Amnis Corp.). The images were compensated for fluorescent dye overlap by using single-stained controls. Gating strategy for CD8-CD4 T cell conjugates analysis is based on the manufacturer's protocol and it was performed as follows: the aspect ratio vs. the area of the bright field image was plotted to identify conjugates. Only conjugates that contained two cells; one cell positive for both CD8 and DAPI and one cell DAPI-positive were considered as true conjugates. Further confirmation of the gated conjugates was done for each CD8-CD4 conjugated T cell by direct observation and photography using the ImageStream. Gating based on CD4 staining was not consistent and it was therefore not used in our gating strategy.

### CD4 T cells apoptosis analyzed by the imagestream

For the apoptosis assay, the conjugates were incubated for 120 min at 37°C and then fixed and permeabilized. Apoptosis was evaluated using both a DNA fragmentation TUNEL assay (BioVision, Apo-BrdU-Red™ K404) and labeling with anti-human active capase-3 antibody (rabbit polyclonal, Enzo ALX-210-807). The cells were also stained with anti-human CD8 antibody conjugated to PerCP/Cy5.5 (clone HIT8a, Biolegend), anti-human CD4 antibody conjugated to PE/Cy7 (clone OKT, Biolegend) and 1.4 μM DAPI (Enzo, BML-AP402-0010). A total of 50,000 cells were analyzed in the ImagStream: those that were CD8-negative, DAPI-positive and positive for either caspase-3 active or TUNEL assay were defined as CD4 T cells in apoptosis.

### Internalization of the ASK1 peptide into CD4 T cells

ASK1 amino acid sequence 152-DEVGEANN-159 that was shown to interact with the Nef protein (21) was synthesized and HPLC purified to 95.07%.(Genemed Synthesis Inc.). 4 × 10^6^ CD4 T cells procured from patients on ART and matching healthy controls were incubated in RPMI supplemented with 1% penicillin/streptomycin and 10% FBS in 10 cm plates. Cells from healthy controls or from patients on ART were incubated with or without 10 μM ASK1 peptide for 16 h at 37°C in 5% CO2 (21). Following incubation, the CD4 T cells were harvested and mixed with equal numbers of autologous CD8 T cells for the apoptosis assay.

### Sample size

The sample size was calculated from the percent conjugated CD4 T cells means and standard deviations reported in our previous study for acute and chronic untreated patients (5), which were found to be similar to the results obtained in the present study. The type 1 error probability was α = 0.05 and the power (1–β) was 0.90. This result was obtained in a minimum sample size of 3 patients at each different disease stage. In patients on ART, which are in the focus of the present study the sample size was 12.

### Statistical analysis

Differences between HIV patients and controls and between HIV patient subgroups were compared using a Student *t*-test (for comparison of two groups) or a one-way ANOVA (for comparison of more than two groups). Differences were considered significant when *p* ≤ 0.01(^*^) and very significant when *p* ≤ 0.001 (^**^). All calculations were done using SPSS v15.0 by IBM and MedCalc V14.8 by MedCalc software. Values are presented as mean ± standard error of the mean.

## Results

### HIV DNA observed in CD4 T cells interacting with autologous CD8 T cells throughout the course of HIV infection

**Figure 1:**
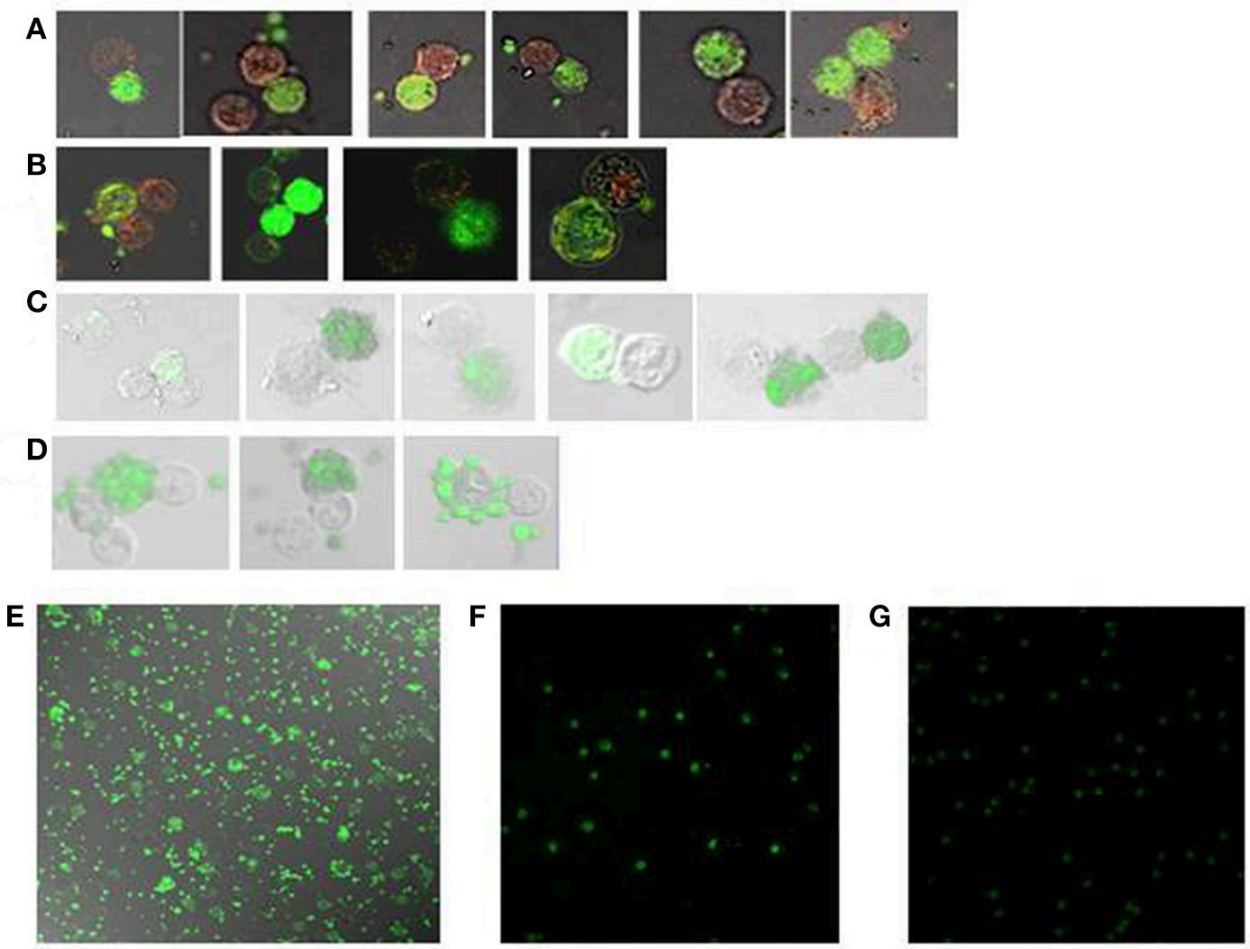
HIV DNA observed in CD4 T cells interacting with autologous CD8 T cells throughout the course of HIV infection. Following incubation of CD4 T cells with autologous CD8 T cells *in situ* PCR of HIV LTR DNA was performed, followed by hybridization with a 56nt long FITC-labeled DNA probe (green). Anti-perforin antibody labeled by Alexa Fluor 647 (red) was used for immunohistochemistry **(A, B)**. The slides were then observed by confocal microscopy (x63). CD4 T cells were conjugated by autologous CD8 T cells, as observed in: **(A)** acute HIV infection, **(B)** chronic HIV infection, **(C)** a chronic HIV-infected patient on ART, **(D,E)** an AIDS patient, **(F)** CD4 T cells from an AIDS patient incubated without CD8 T cells, **(G)** a healthy control.

We studied CD4 T cells conjugated with autologous CD8 T cells procured from the PBMC of AIDS, acute, and chronic untreated and treated HIV-infected patients as well as healthy controls (Tables 1, 2; Figure 1). The CD4 and CD8 T cells were procured directly from the PBMC of HIV-infected patients by means of magnetic beads, followed by co-incubation and fixation on slides. The cells were neither manipulated nor stimulated *in vitro. In situ* PCR was performed on the fixed cells by means of primers from the HIV long terminal repeat (LTR). The same primers were used for the identification of HIV provirus in CD4 T cells in various studies (27–29). The probe was a conserved sequence of 56nt labeled by FITC (5). Anti-perforin APC-labeled antibodies were used after the *in*-*situ* PCR to look for the perforin content in the CD8 T cells.

**Table 2:** Quantification of HIV DNA-positive CD4 T cells and CD4-CD8 T cell conjugates throughout the course of HIV infection observed by *in*-*situ* PCR.

**HIV Infection**	**Acute**	**Chronic untreated**	**Chronic on ART**	**AIDS**
**Number of patients**	3	3	12	3
**HIV-DNA-positive CD4 T cells, %**	11 ± 3.0	10 ± 2.0	12 ± 1.5	9.5 ± 2.0
**Conjugated CD4 T cells, %**	6.5 ± 1.0	3 ± 0.6	3 ± 0.3	7.0 ± 0.9
**Conjugated HIV-DNA-positive CD4 T cells**, %	70 ± 3.0	30 ± 5	25 ± 3.0	75 ± 8.0

HIV DNA was detected in ~10% of CD4 T cells procured from all stages of HIV infection. From 25 to 30% of the HIV-infected CD4 T cells were conjugated by CD8 T cells in chronic untreated patients and patients on ART, which are 3 ± 0.6 and 3 ± 0.3% of the CD4 T cells, respectively (Table 2; Figures 1B,C). In contrast, from 70 to 75% of the HIV-infected CD4 T cells were conjugated by autologous CD8 T cells in acute HIV infection and AIDS patients, which are 6.5 ± 1.0 and 7.0 ± 0.9% of the CD4 T cells, respectively (Table 2; Figures 1A,D). The CD4 T cells procured from healthy controls were negative for HIV DNA (Figure 1G). The conjugation results for acute and chronic untreated HIV-infected patients as observed by *in situ* PCR were similar to those observed previously by directly interacting CD8 T cells with calcein-labeled CD4 T cells (5). Calcein-labeled CD4 T cells and autologous CD8 T cells procured from the PBMC of AIDS patients were co-incubated and observed directly by fluorescent microscopy: similar to the *in-situ* PCR results (Table 2), 7.0 ± 0.65 % of the CD4 T cells were conjugated by CD8 T cells compared to 0.9 ± 0.07% of the cells of the matched healthy controls.

CD4 T cells and autologous CD8 T cells procured from AIDS patients and co-incubated revealed robust apoptotic morphology of the HIV-infected CD4 T cells with numerous apoptotic bodies (Figures 1D,E). In contrast, the CD4 T cells that were incubated without the autologous CD8 T cells demonstrated no apoptotic morphology (Figure 1F). Very rare apoptotic events were observed in HIV-infected CD4 T cells incubated with autologous CD8 T cells of chronic untreated HIV-infected patients and patients on ART. It is worth noting that in an earlier study (5) with 11 chronic untreated HIV patients the conjugation and apoptosis rates were observed with wide range of viral loads (189-96,389 copies/ml). Hence the copy by itself had no influence on the outcome of conjugation and lysis.

### Interaction of CD8 and autologous CD4 T cells in HIV-infected patients on ART

**Figure 2:**
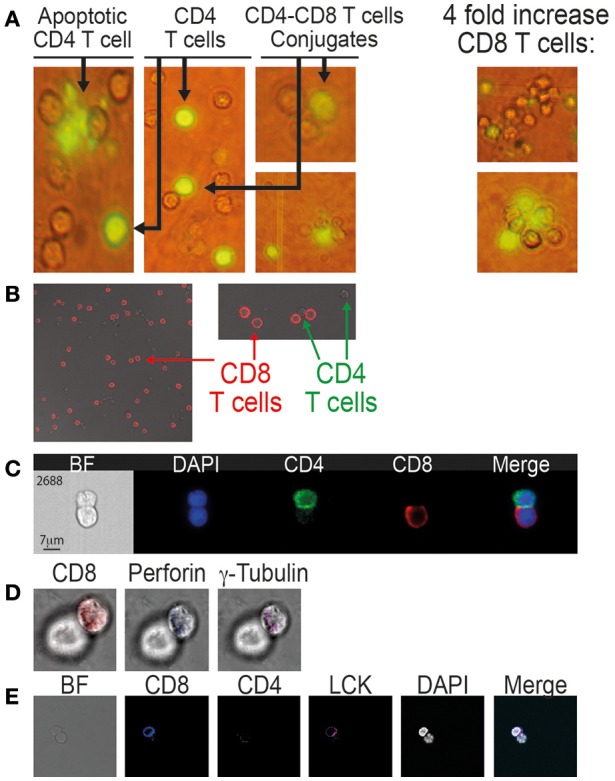
Conjugation and immunological synapse formation between CD8 T cells and autologous CD4 T cells procured from patients on ART. **(A)** Calcein-labeled CD4 T cells co-incubated with autologous CD8 T cells as observed by fluorescent microscopy (x40). **(B)** CD8-CD4 T cell conjugates labeled with anti-CD8 antibody (APC, red) and observed by confocal microscopy (x63). **(C)** CD8-CD4 T cell conjugates labeled with anti-CD8 antibody (APC, red) and anti-CD4 antibody (PE-Cy7, green) and observed by the ImageStream. **(D)** Immunological synapse formation between CD8 and CD4 T cells. The conjugates were labeled with: anti-CD8 antibody, anti-perforin antibody and anti-γ tubulin antibody. **(E)** anti-CD8 antibody, anti-CD4 antibody and anti-LCK antibody.

CD4 T cells procured from seven HIV-infected patients on ART with undetectable viral load were labeled with calcein, incubated with an equal number of autologous CD8 T cells, and screened by fluorescence microscopy: 2.3 ± 0.3% of the CD4 T cells were conjugated by autologous CD8 T cells compared to 0.8 ± 0.1% in the healthy controls. The four-fold increase of the CD8 T cells interacting with the CD4 T cells resulted in an increase of the conjugation: 5.7 ± 1.3% compared to 1.2 ± 0.3% in the healthy controls (Table 3; Figure 2A). The CD4-CD8 T cells interaction was further quantified after labeling the CD8 T cells with anti-CD8 antibody (APC labeled), and the results showed that 3% of the CD4 T cells were conjugated by the CD8 T cells (Figure 2B). Calcein-labeled CD4 T cells interacting with non-autologous CD8 T cells procured from patients on ART resulted in only 1 ± 0.2 % conjugation, similar to the healthy controls.

**Table 3:** Conjugation of CD8 T cells with autologous CD4 T cells procured from chronic HIV-infected patients on ART.

**Method**	**CD4:CD8 T cell ratio**	**Sample type**	**Number**	**%Conjugation**
Calcein-labeled CD4 T cells interacting with CD8 T cells	1:01	Patients on ART	7	2.3 ± 0.3
		Controls	7	0.8 ± 0.1
	1:04	Patients on ART	3	5.7 ± 1.3
		Controls	3	1.2 ± 0.3
CD4/CD8 T cells observed by ImageStream	1:01	Patients on ART	5	2.5 ± 0.5
		Controls	5	0.9 ± 0.2
	1:04	Patients on ART	2	4.8 ± 0.4
		Controls	2	1.4 ± 0.3

Multi-spectral imaging flow cytometer (ImagStream, Amnis) was used to study the conjunction of CD8 T cells with autologous CD4 T cells procured from an additional five patients on ART and compared to matched healthy controls. The ImageStream combines flow cytometry with single-cell microscopy for the analysis and quantification of fluorescent signals for thousands of cells per sample. Following conjugation, the cells were fixed by 4% PFA and labeled with anti-CD4 antibody (PE/Cy7-labeled), anti-CD8 antibody (PerCP/Cy5.5 labeled) and DAPI. A total of 50,000 cells from each sample were analyzed. The gating strategy to identify and quantify the conjugates is based on the aspect ratio plotted against the area and it was confirmed by directly observing the selected cell populations. Each selected conjugate was observed and photographed by the ImageStream (Figure 2C). By the ImageStream analysis 2.5 ± 0.5% of the CD4 T cells procured from patients on ART were conjugated with autologous CD8 T cells compared to 0.9 ± 0.2% in the healthy controls. Four-fold increases of the CD8 T cells interacting with CD4 T cells resulted in an increase in the conjugation of the CD4 T cells to 4.8 ± 0.4% compared to 1.4 ± 0.3% in the healthy controls (Table 3).

The CD4-CD8 T cell conjugates from patients on ART were studied to identify known specific immunological synapse proteins (30). After the conjugation of CD4 T cells with autologous CD8 T cells, the cells were fixed (4% PFA), permeabilized and labeled with anti-CD8, anti γ-tubulin, and anti-perforin antibodies (Figure 2D). Additionally, the synapse was studied by anti-CD4, anti-CD8, and anti-LCK antibodies (Figure 2E). Both γ-tubulin and perforin localized in the interface between the CD8 T cell and the conjugated CD4 T cell. It has been reported that γ-tubulin, the centrosome, polarizes toward the synapse and docks at the plasma membrane once the TCR is engaged (30). Perforin expression indicates activation and killing capacity of CD8 T cells, and perforin co-localizes with granzyme B in cytotoxic granules that are polarizing to the immunological synapse (3, 31). LCK, which is a key protein tryosine kinase that initiates intracellular signaling by the TCR (32), was also observed at the synapse between CD8 and CD4 T cells procured from HIV-infected patients on ART (Figure 2E).

### Apoptosis of CD4 T cells caused by autologous CD8 T cells procured from HIV-infected patients on ART

**Figure 3:**
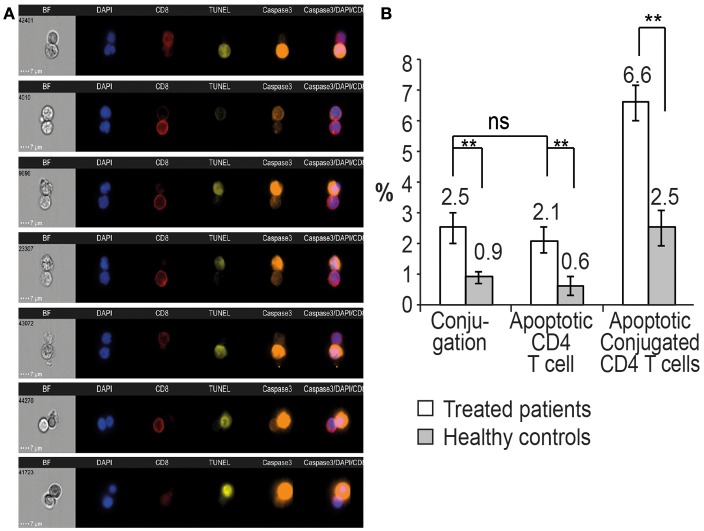
Conjugation and apoptosis of CD4 T cells by autologous CD8 T cells procured from patients on ART and analyzed by the ImageStream. **(A)** Apoptosis assay of CD4 T cells with CD8 T cells procured from a 43-year-old female patient on ART with undetectable viral load and a CD4 T cell count of 507 cells/mm^3^ (39%). The CD4 T cells in apoptosis are positive for activated caspase 3 (orange) and DNA fragmentation, TUNEL assay (yellow), CD8 T cells (red), DAPI (purple). **(B)** Quantitative analysis of conjugation and apoptosis of CD4 and CD8 T cells in five patients on ART compared to five healthy controls. ** indicate *P*-values <0.01, two-tailed paired *t*-test.

CD4 T cells procured from HIV-infected patients on ART or healthy controls were mixed with equal number of autologous CD8 T cells and incubated at 37°C for 120 min (5). The cells were then fixed with 4% PFA, permeablized, and labeled with anti-CD8 antibody (APC-labeled) and the apoptotic markers: anti-active caspase-3 antibody (AF568-labeled) and terminal deoxynucleotidyl transferase dUTP nick end labeling (TUNEL) assay (Ex/Em = 488/576). Apoptosis was defined as being positive by the gating of one or both of the apoptotic markers. Conjugation was quantified in the ImageStream by gating the aspect ratio vs. area of the bright field image and identifying both CD4 and CD8 signals in the conjugates. A total of 50,000 cells from each sample were analyzed both by FACS and by single-cell microscopy. Figure 3A demonstrates an apoptotic assay of autologous CD8 and CD4 T cells procured from a 43-year-old female patient who had been on ART for over 10 years and who had an undetectable viral load and CD4 T cells of 507 cells/mm^3^ (39%). Both apoptotic markers, caspase-3 activation (orange) and DNA fragmentation by TUNEL assay yellow), can be seen in the CD4 T cells conjugated with autologous CD8 T cells.

Quantitative analysis by the ImageStream of conjugation and apoptosis of CD4 T cells in five patients on ART was performed and compared to matched healthy controls. Apoptotic CD4 T cells comprised 2.1 ± 0.4% of the CD4 T cells, compared to 0.6 ± 0.3% of the cells in the healthy controls. Additionally, 2.5 ± 0.4% of the CD4 T cells were conjugated by the CD8 T cells. Only 6.6% of the conjugated CD4 T cells were positive for the apoptotic markers compared to 2.5 ± 0.6% of the conjugated cells in the healthy controls (Figure 3B), suggesting a possible inhibition of apoptosis in the HIV-infected CD4 T cells.

### Latent reservoir CD4 T cells can be conjugated by autologous CD8 T cells which may lead to their apoptosis

**Figure 4:**
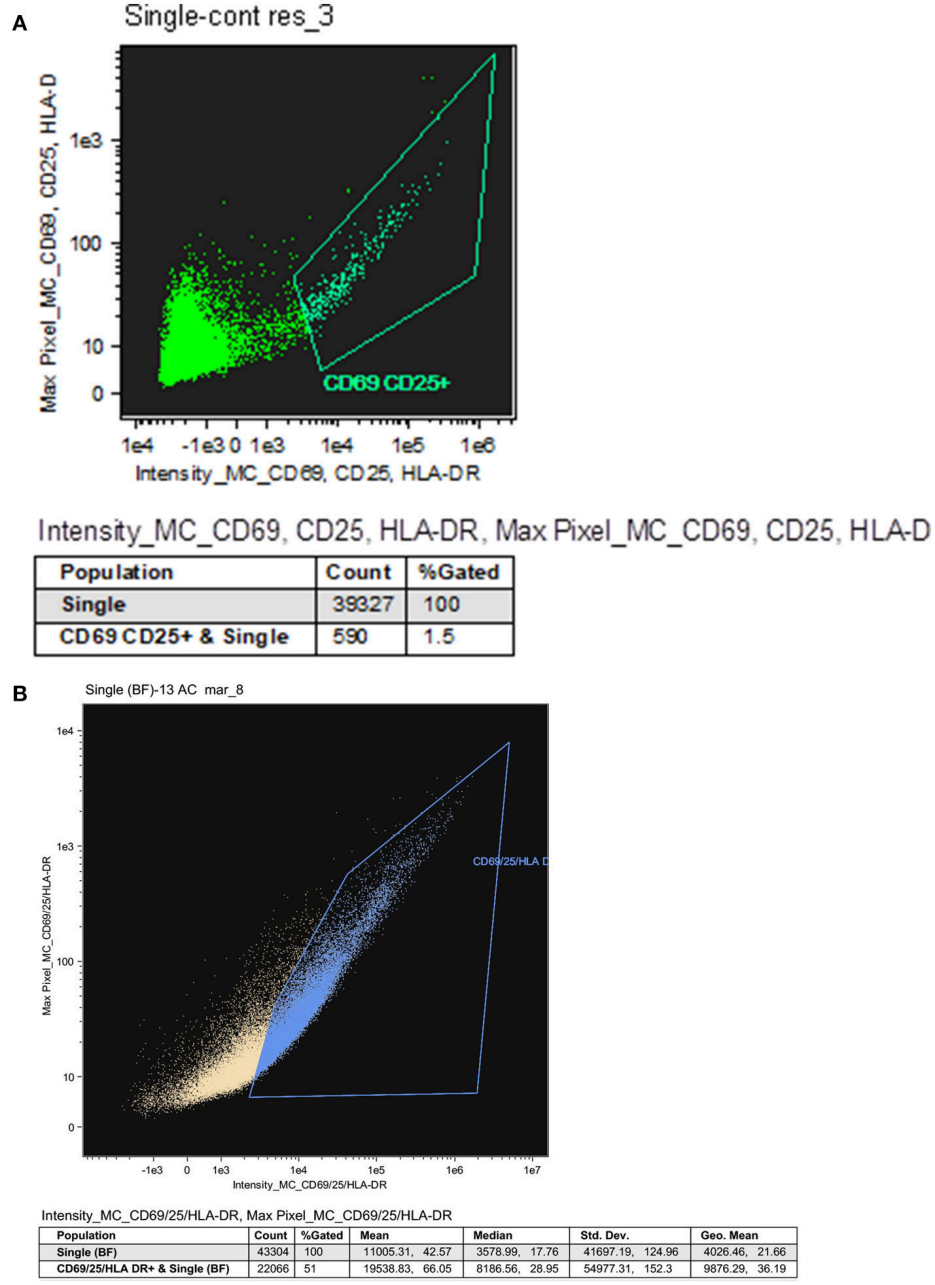
Resting CD4 T cells procured from HIV infected patients on ART. **(A)** Representative analysis indicating the purity of resting CD4 T cells. Resting CD4 T cells procured from the PBMC of HIV infected patients on ART were purified using a two-step negative selection, stained with anti-human CD25, anti-human CD69 and anti-human HLA-DR antibodies and analyzed by the ImageStream. **(B)** The fraction of resting and activated CD4 T cells. CD4 T cells procured from the PBMC of HIV infected patients on ART were labeled with antibodies against CD25, CD69 and HLA-DR followed by ImageStream analysis. 51% of the CD4 T cells were CD25, CD69, and HLA-DR positive (activated cells) and 49% were CD25, CD69, and HLA-DR negative (resting cells).

HIV persists in both treated and untreated infected patients in a stable pool of resting CD4 T cells as a latent but replication competent provirus (2). These proviruses can express some but not all gene products in the absence of virion production (33–35). To study the resting memory CD4 T cells population, we separated resting from activated CD4 T cells procured from HIV-infected patients both untreated and treated by ART by a published two-step bead depletion procedure (22, 24). After separating the CD8 T cells using magnetic beads, the CD4 T cells were negatively selected using magnetic beads coated with a cocktail of monoclonal antibodies (Miltenyi CD4 T cell isolation kit). The purified CD4 T cells were further separated into resting and activated cells by beads coated with CD69, CD25, and HLA-DR antibodies as the activated CD4 T cell population. The CD69, CD25, and HLA-DR negative cells are the resting memory cells. The purity of the resting memory CD4 T cells was routinely assessed by staining them with antibodies against CD69, CD25, and HLA-DR and analyzing 50,000 cells using the ImageStream. Typical purities of resting CD4 T cells were 95–98.5% with <1.5% contamination by activated CD4 T cells (Figure 4A). The fraction of resting and activated CD4 T cells before cell separation was evaluated by staining CD4 T cell samples with APC-labeled antibodies against CD69, CD25, and HLA-DR followed by ImageStream analysis of 50,000 cells. Activated cells (CD25, CD69, and HLA-DR positive) were 51% of the CD4 T cells and 49% were resting cells (CD25, CD69, and HLA-DR negative; Figure 4B).

**Figure 5:**
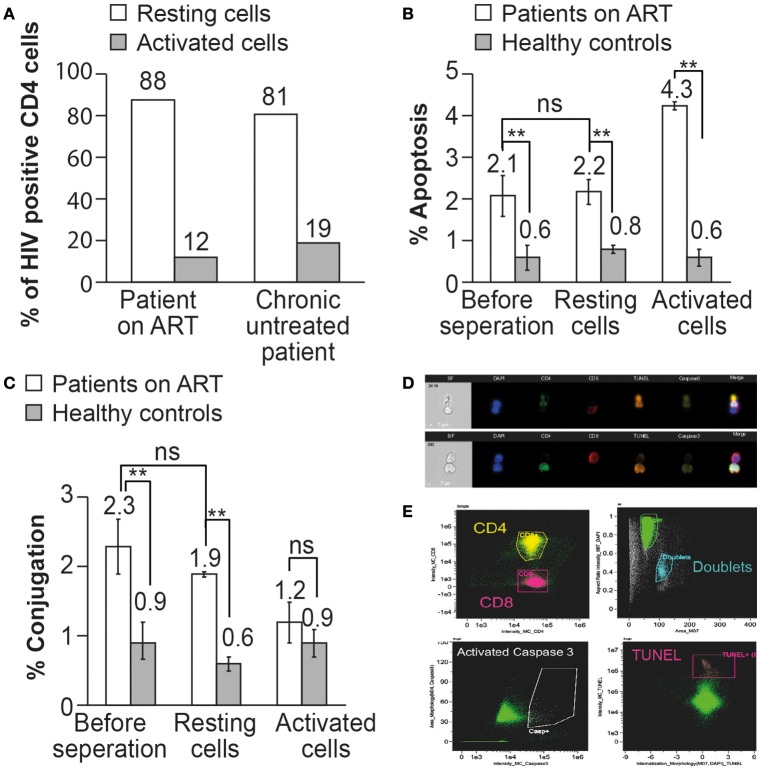
The resting memory CD4 T cells can be recognized and undergo apoptosis by autologous CD8 T cells in patients on ART. **(A)** *In situ* PCR was performed on resting and activated CD4 T cells procured from an untreated HIV-infected patient and from a patient on ART. The percent of HIV-infected CD4 T cells was calculated out of 1000 resting or activated CD4 T cells. **(B)** The apoptosis of un-separated, resting, and activated CD4 T cells by autologous CD8 T cells was analyzed in 3 patients on ART by ImageStream analysis. The apoptotic cells were gated and observed by anti-activated caspase 3 antibody and TUNEL assay. **(C)** The conjugation of CD8 T cells with autologous CD4 T cells before or after separation to resting or activated cells was analyzed in 3 patients on ART by ImageStream analysis. The conjugates were gated by plotting the aspect ratio against the area and the results were confirmed by directly observing the selected cell populations. **(D)** Representative images obtained by the ImagStream analysis of resting memory CD4 T cells conjugated by autologous CD8 T cells undergoing apoptosis. CD4 in green, CD8 in red, TUNEL in orange, and activated caspase 3 in yellow. **(E)** Representative imaging flow cytometry plots of resting memory CD4 T cells conjugated by autologous CD8 T cells undergoing apoptosis. **P < 0.01, as obtained in two-tailed paired *t*-test.

*In-situ* PCR of HIV DNA was performed on resting and activated CD4 T cells procured from a 52-year-old male patient on ART and a 55-year-old male untreated patient. A total of 1000 cells were screened in the *in situ* PCR slides by confocal microscopy. Four percent of the activated CD4 T cells of the patient on ART contained HIV DNA and 5% of the activated CD4 T cells in the untreated patient contained HIV DNA. In contrast, most of the HIV DNA was found in the resting memory CD4 T cells: 30% in the patient on ART and 22% in the untreated patient. Our results indicate that 88 and 81% of the CD4 T cells containing HIV DNA are resting memory cells as opposed to 12 and 19% of the activated CD4 T cells in the patient on ART and in the untreated patient, respectively (Figure 5A). In those patients, the ImageStream analysis revealed that about 50% of the CD4 T cells were activated and that 50% of them were resting, findings that are comparable to published data in their age group (36).

Conjugation and apoptosis of resting and activated CD4 T cells by autologous CD8 T cells procured from three patients treated by ART and three healthy controls were analyzed by the ImagStream (Figures 5B–D). Following the separation of the activated and resting CD4T cells, they were incubated with autologous CD8 T cells for 2 h (5) and then fixed by 4% PFA and stained with anti-CD4 antibody, anti-CD8 antibody, anti-active caspase-3 antibody, and DNA fragmentation assay (TUNEL assay). ImageStream analysis was performed on 50,000 cells from each sample. Conjugation of resting CD4 T cells with autologous CD8 T cells was comparable to un-separated CD4 T cells (1.9 ± 0.3 and 2.3 ± 0.3%, respectively). The conjugation of the activated CD4 T cells was not significantly different from that of the control (1.2 ± 0.2 and 0.9 ± 0.2%, respectively), probably due to the very low content of HIV DNA in the activated CD4 T cells as demonstrated by *in situ* PCR (Figure 5A).

The apoptosis of the total CD4 T cells was comparable with that of the resting CD4 T cells after incubation with autologous CD8 T cells (2.2 ± 0.4 and 2.1 ± 0.5%, respectively), at levels significantly higher than those in healthy controls (0.6 ± 0.2 and 0.8 ± 0.3%, respectively). The activated CD4 T cells after incubation with autologous CD8 T cells demonstrated significantly higher apoptosis (4.3% ± 0.3) than that of the resting cells, indicating that activated CD4 T cells are more susceptible to CD8 T cell-induced apoptosis. The ImageStream clearly demonstrated that the resting memory cells can be conjugated by autologous CD8 T cells and undergo apoptosis (Figures 5D,E).

### Inhibition of HIV Nef interaction with ASK1 in the CD4 T cells procured from patients on art results in increased susceptibility to killing by autologous CD8 T cells

HIV Nef protein interacts with ASK 1 and inhibits its pro-apoptotic death signaling by Fas/FasL and TNFα, thus protecting HIV-infected cells from being killed by CD8 T cells (19). A peptide derived from ASK1 protein sequence, amino acids 152 to 159 (DEVGEANN) inhibited the interaction between Nef and ASK1 and circumvented the anti-apoptotic effect of Nef, restoring ASK1 pro-apoptotic function induced by TNFα (21). The possible role of HIV Nef protein in protecting the latent reservoir cells from being killed by CD8 T cells was investigated by using the peptide (DEVGEANN). CD4 T cells and autologous CD8 T cells were procured from the PBMC of four HIV-infected patients on ART and matched healthy controls. The CD4 T cells were incubated with 10 μM peptide (DEVGEANN) for 16 h at 37°C in 5% CO2 (20). CD4 T cells procured from the same patients on ART and healthy controls were incubated without the peptide as a control. Following the incubation, the CD4 T cells were mixed with autologous CD8 T cells and incubated for 120 min at 37°C for the cytotoxic assay. The cells were then fixed by 4% PFA and labeled with anti-CD4 antibody, anti-CD8 antibody, anti-active caspase-3 antibody, and DNA fragmentation assay (TUNEL assay). Between 10,000 and 50,000 cells from each sample were analyzed by the ImageStream.

**Figure 6:**
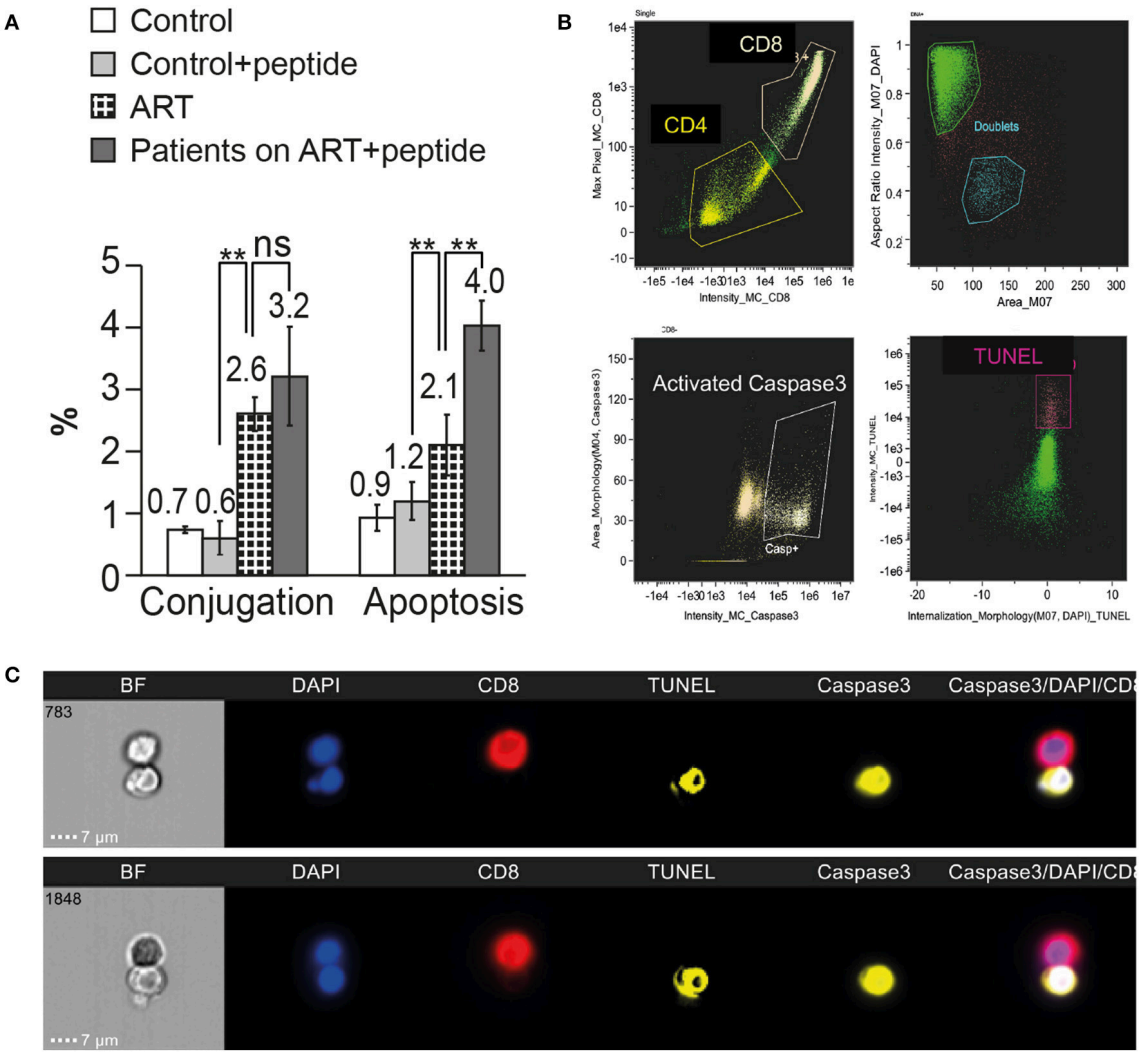
Inhibition of HIV Nef interaction with ASK1 in the CD4 T cells procured from patients on ART results in increased susceptibility to killing by autologous CD8 T cells. CD4 T cells and autologous CD8 T cells were procured from the PBMC of four HIV-infected patients on ART and matched healthy controls. The CD4 T cells were incubated with ASK1 peptide that interferes with Nef-ASK1 interaction. Following the incubation with or without the peptide, the CD4 T cells were mixed with autologous CD8 T cells for the cytotoxic assay. The cells were then fixed and labeled with anti-CD4 antibody, anti-CD8 antibody, anti-active caspase-3 antibody and DNA fragmentation assay (TUNEL assay) and analyzed by the ImageStream. **(A)** Four independent conjugation and apoptosis assays are presented. ** indicate *P*-values < 0.01, as obtained by two-tailed paired *t*-test. **(B)** Representative imaging flow cytometry plots of CD4 T cells incubated with the peptide and interacted with CD8 T cells. **(C)** ImageStream images of TUNEL and activated caspase-3 positive CD4 T cells treated with ASK1 peptide conjugated with autologous CD8 T cells.

The incubation with the peptide did not affect the conjugation between the CD4 and CD8 T cells both in HIV-infected patients on ART (2.6 ± 0.3 % without the peptide and 3.2 ± 0.8% with the peptide) and healthy controls (0.7 ± 0.1 without the peptide and 0.6 ± 0.3 with the peptide; Figure 6A). The apoptosis of the CD4 T cells incubated with the peptide was significantly higher than the apoptosis of CD4 T cells without the peptide (4.0 ± 0.4 and 2.1 ± 0.5%, respectively). The peptide did not have any effect on the apoptosis of CD4 T cells procured from healthy controls 0.9 ± 0.2% without the peptide and 1.2 ± 0.3% with the peptide.

## Discussion

HIV infection is currently treatable but not curable because anti-retroviral therapy (ART) suppresses HIV viral load without affecting the viral “latent reservoir.” The role of CTL in the course of HIV infection and the way they affect the virus that resides in the latent reservoir is unknown. There are ~10% of HIV-specific CD8 T cells in the peripheral blood mononuclear cells (PBMC) of HIV-infected patients (12) and their potential targets HIV-infected CD4 T cells (17, 25, 37). We investigated the interaction between CD8 T cells and autologous CD4 T cells procured from the blood of HIV-infected patients. The cells were neither manipulated nor stimulated *in vitro*. We had previously shown that PBMC of acute and chronic HIV-infected patients who were not treated by ART contain HIV-specific CD8 T cells that conjugate with and kill HIV-infected autologous CD4 T cells (5). The results of the present study demonstrate that CD8 T cells conjugate with and kill autologous HIV-infected CD4 T cells throughout the course of HIV infection, including those of patients on ART. The conjugation activity and apoptosis during acute HIV infection and AIDS were much higher compared to chronic HIV-infected patients untreated or treated by ART. We demonstrated that the latent reservoir cells can also be targeted by autologous CD8 T cells. This interaction is of low magnitude and considered as being “controlled” during the chronic stage of HIV infection in patients who are untreated or treated by ART. Our results suggest that the HIV Nef protein plays a role in protecting the latent reservoir cells from the killing of CD8 T cells.

CD8 T cells interacting with autologous CD4 T cells were fixed on slides and studied by *in situ* PCR of HIV DNA. The *in situ* PCR primers (NEC152 and NEC131) are conserved in the HIV LTR sequence and were described before (27, 28, 38). The primers are currently in use by the central virology laboratory of the Israeli Ministry of Health to identify HIV DNA in lymphocytes of newborns from HIV-infected mothers. We used a 56nt probe, which was conserved in the amplified DNA. The LTR is expected to be present in most of the integrated viral DNA, and the method that we used to recognize it should not be affected by the abundance of internal deletions and hypermutations of the described integrated virus (39). HIV DNA was demonstrated in about 10% of the CD4 T cells obtained from acute infected patients, chronic untreated infected patients, patients on ART, and AIDS patients (Table 1). Comparable results using *in situ* PCR were also reported by others: Patterson et al. used *in situ* PCR and showed that 9.3 ± 3.3% of the PBMC of 9 chronic untreated HIV infected patients have amplified HIV DNA within them (37). Bagasra et al. reported on HIV DNA in the PBMC procured from chronic infected patients and AIDS patients: the HIV-positive cells ranged between 0.1 and 13.5% of all cells (average 4.3 ± 3.0%) (25, 40). A method that quantify the *in-situ* PCR product by flow cytometry was developed by Re et al. They reported that the frequency of HIV infected CD4 T cells in the PBMC range from 0.6 to 20% (29, 41, 42). The frequency of HIV-infected CD4 T cells obtained by the *in situ* PCR method is in agreement with the conjugation frequency observed between CD8 T cells and autologous CD4 T cells during the various stages of HIV infection reported here and by Chorin et al. (5).

We separated active CD4 T cells (CD69-, CD25-, and HLA-DR-positive) from resting memory CD4 T cells (CD69-, CD25-, and HLA-DR-negative) procured from chronic untreated and treated HIV-infected patients and assayed them by *in situ* PCR of HIV DNA. We found that the majority of the HIV-infected CD4 T cells in those patients reside in the resting memory (latent reservoir) cell compartment. There was no decline of HIV-infected CD4 T cells in patients on ART, indicating a high level of stability of the latent reservoir cells even in the absence of viral replication (1).

Formation of CD8-CD4 T-cell conjugates and apoptosis of the CD4 T cells were observed by fluorescence microscopy, imaging flow cytometry (ImageStream) and *in situ* PCR of HIV DNA of the conjugated CD4 T cells (Tables 2, 3; Figure 2). In addition to a high conjugation rate, there was robust apoptosis of the HIV-positive CD4 T cells after incubation with autologous CD8 T cells in AIDS patients (Figure 1). In contrast, the conjugation and apoptosis rates were much lower in the chronic untreated and ART-treated HIV-infected patients. Similar results were observed in studies of acute compared to chronic untreated HIV infection where conjugation and apoptosis were significantly higher in acute HIV-infected patients (5). These results suggest a possible inhibition of conjugation and apoptosis of the CD4 T cells interacting with autologous CD8 T cells in chronic HIV-infected patients and patients on ART. The apoptotic activity is apparently unleashed in AIDS as well as in acute HIV infection.

At present, all the patients infected by HIV are treated by ART, and most of them have an undetectable viral load. Markers of inflammation have been documented in those patients, suggesting continuous response of the immune system against the latent reservoir cells (43). The latent reservoir cells are not affected by treatment with ART, and the virus rebounds rapidly once treatment is stopped. We found continuous interaction of CD8 T cells with autologous CD4 T cells in patients on ART. Conjugation of CD8 with CD4 T cells was measured and found to be similar by three different assays: *in situ* PCR of HIV DNA in the CD4 T cells (Table 1; Figure 1), calcein-labeled CD4 T cells conjugation assay and ImageStream analysis of conjugated cells marked by antibodies (Table 2; Figure 2). The presence of an active immunological synapse between the CD8 and the autologous CD4 T cells was confirmed using specific markers of an immunological synapse that were observed with confocal microscopy (Figure 2). A significant level of apoptosis of the CD4 T cells following incubation with autologous CD8 T cells procured from patients on ART was observed by the ImageStream (Figure 3).

Our results demonstrate for the first-time ongoing interaction between CD8 and autologous CD4 T cells even in HIV-infected patients on ART. Assuming that the interaction is between CD8 T cells and the latent reservoir CD4 T cells, highly purified resting CD4 T cells were isolated from the PBMC of patients on ART using a negative depletion isolation method (22, 24). Conjugation and apoptosis of resting memory CD4 T cells by autologous CD8 T cells were comparable to unseparated CD4 T cells, as observed by ImageStream analysis (Figures 5B,C), suggesting that the activity of the CD8 T cells is against latent reservoir CD4 T cells.

The biology of latent reservoir CD4 T cells is still a mystery. We observed interactions between CD8 T cells and autologous CD4 T cells that contain HIV DNA within them. We assume that CD8 T cells recognize HIV epitopes presented by the HLA receptor of the resting memory CD4 T cells. Ho et al. analyzed the structure of integrated HIV DNA in latent reservoir cells and found that 11.7% of them consist of the whole viral genome, with the majority consisting of large deletions and hypermutations (39). Fromentin et al. reported expression of unspliced HIV RNA in CD4 T cells in patients on ART (44). Imamichi et al. found defective HIV proviruses that produce novel coding RNA species involving elements of gag, pol, env, rev, and nef maintaining translationally competent open reading frames in HIV-infected patients on ART (45). Pace et al. documented that resting cells that had been spinoculated by w/ NL4-3 *in vitro* were capable of producing HIV Gag protein without supporting productive infection (35). Using a primary cell model of latency Thomas et al. found that a Nef-specific CD8 T-cell clone exhibited low-level recognition of this latently HIV infected cells prior to their reactivation. Moreover, they observed Nef specific CD8 T cell response in the PBMC of ART treated patients, which indicate an ongoing expression of Nef during prolong ART treatment (15). This data can suggest that low-grade viral proteins are expressed and presented by resting memory CD4 T cells that are recognized by CD8 T cells.

We found significant apoptosis in acute HIV infection and AIDS, while patients in chronic stages of HIV infection and patients on ART had low-grade apoptotic activity of the CD4 T cells, including resting memory cells. Such low-grade apoptotic activity was observed even in the conjugated CD4 T cells (Figure 3B). The HIV Nef protein interferes with CTL activity by down-regulating HLA expression, expressing FasL molecules on the surface of the HIV-infected CD4 T cells and protecting the HIV-infected CD4 T cells from the killing of CTL by the interaction of Nef with ASK1 protein (19, 20). Infection of rhesus macaques with Nef-defective SIV resulted in low viral load and caused a substantial delay in the onset of disease (46). Rare individuals infected by HIV containing Nef deletion are observed to have low viral loads and stable CD4 T cell counts for many years even in the absence of treatment by ART (47). Inhibition of Nef interaction with ASK1 was designed to study the possibility that HIV Nef protein is involved in the inhibition of apoptosis in the CD4 T cells. Kumar et al. identified the domain in the ASK1 that interacts with HIV Nef protein. This 8-amino acid peptide inhibited ASK1-Nef interaction and abolished the Nef protection of cells from apoptosis induced by tumor necrosis factor α (TNFα) (21). We found that the delivery of the peptide to the CD4 T cells before the interaction with autologous CD8 T cells resulted in a 2-fold increase of the apoptosis of the CD4 T cells in the apoptotic assay (Figure 6). These data suggest that the virus keeps the latent reservoir CD4 T cells protected from the immune system by expressing HIV Nef protein. Viral proteins that interfere with CTL activity resulting in the maintenance of latency were described in herpes viruses, adenoviruses, cowpox virus, vaccinia virus, and HTLV-1. Similar to Nef activity, these proteins act by down-regulating HLA expression, interfering with the virally infected cellular apoptotic machinery and manipulating Fas/FasL interaction (18, 48). Immune checkpoint molecules, PD-1, TIGIT and LAG-3, are positively associated with the frequency of CD4 T cells harboring integrated HIV DNA in patients on ART. This serves as evidence for continuous interaction with and evasion from the CTL in patients on ART (43, 48). Further studies are needed to understand the viral and cellular mechanisms that up-regulate those immune checkpoints in latently infected HIV CD4 T cells.

Mathematical modeling of CTL activity during HIV infection is consistent with lytic control by CTL, and predicts that most productively infected cells are killed by CTL (10, 49). Apart from their positive effects of controlling viral spread, CTL have been shown to cause tissue damage in viral infections, such as in viral myocarditis (50) and viral hepatitis. We propose that CD4 T cell annihilation in HIV-infected patients results from the ongoing interaction of CD8 T cells with HIV-infected CD4 T cells. We assume that a dynamic balance is established between HIV-infected CD4 T cells and HIV-specific CD8 T cells during the chronic stage of HIV infection, resulting in restrained apoptosis of the CD4 T cells and leading to their gradual decline. The virus manipulates the immune system to maintain a low-grade infection, thus achieving prolonged survival combined with efficient viral spread. We suggest that Nef have a role in maintaining this balance based on our findings that inhibition of Nef resulted in significant increase in the killing of CD4 T cells by autologous CD8 T cells. We propose that CTL activity following Nef inhibition may help eliminate the latently HIV- infected CD4 T cells reservoir. An additional definitive study that includes a larger population of HIV infected patients should be done. In addition, the study of the role of Nef in HIV infected patients could be expended to elucidate the molecular effect of Nef in response to CD8 T cells conjugation.

## Ethics statement

This study was carried out in accordance with the recommendations of the institutional Tel-Aviv Medical center ethics committee in accordance with the ethical standards laid down in the 1964 Declaration of Helsinki and its later amendments. The protocol was approved by the Tel-Aviv Medical center ethics committee. All subjects gave written informed consent in accordance with the Declaration of Helsinki.

## Author contributions

ZS design the research, acquired the data performed the experiments, analyzed the data, performed the statistical analysis and drafted the manuscript. EC design the research, contributed to the interpretation of the results and edited the manuscript. OG-G design the research, contributed to the interpretation of the results. EZ contributed to the interpretation of the results. DT and BA provided samples and contributed to the interpretation of the results. GB design the research and contributed to the interpretation of the results. DH design and oversaw the research, analyzed, and interpreted the data, wrote the manuscript and revised the final version of the manuscript.

### Conflict of interest statement

The authors declare that the research was conducted in the absence of any commercial or financial relationships that could be construed as a potential conflict of interest.

## Abbreviations

PBMC: Peripheral blood mononuclear cells
HIV: Human immunodeficiency virus
LTR: Long terminal repeat
ART: Antiretroviral therapy
TCR: T cell receptor
CTL: Cytotoxic T lymphocyte
HLA: Human leukocyte antigen
MHC: Major histocompatibility complex
TUNEL: Terminal deoxynucleotidyl transferase dUTP nick end labeling.
